# Supplementary material for: Gama rays mediated improvement of catalytic efficiency and thermostability of glucoamylase by replacing active site leucine to isoleucene from super koji (Aspergillus oryzae)
Source: PLoS One. 2025 Apr 18;20(4):e0319261. doi: 10.1371/journal.pone.0319261 (PMC12007714; doi:10.1371/journal.pone.0319261)
Supplement: S1 File — (PDF) [file pone.0319261.s001.pdf]

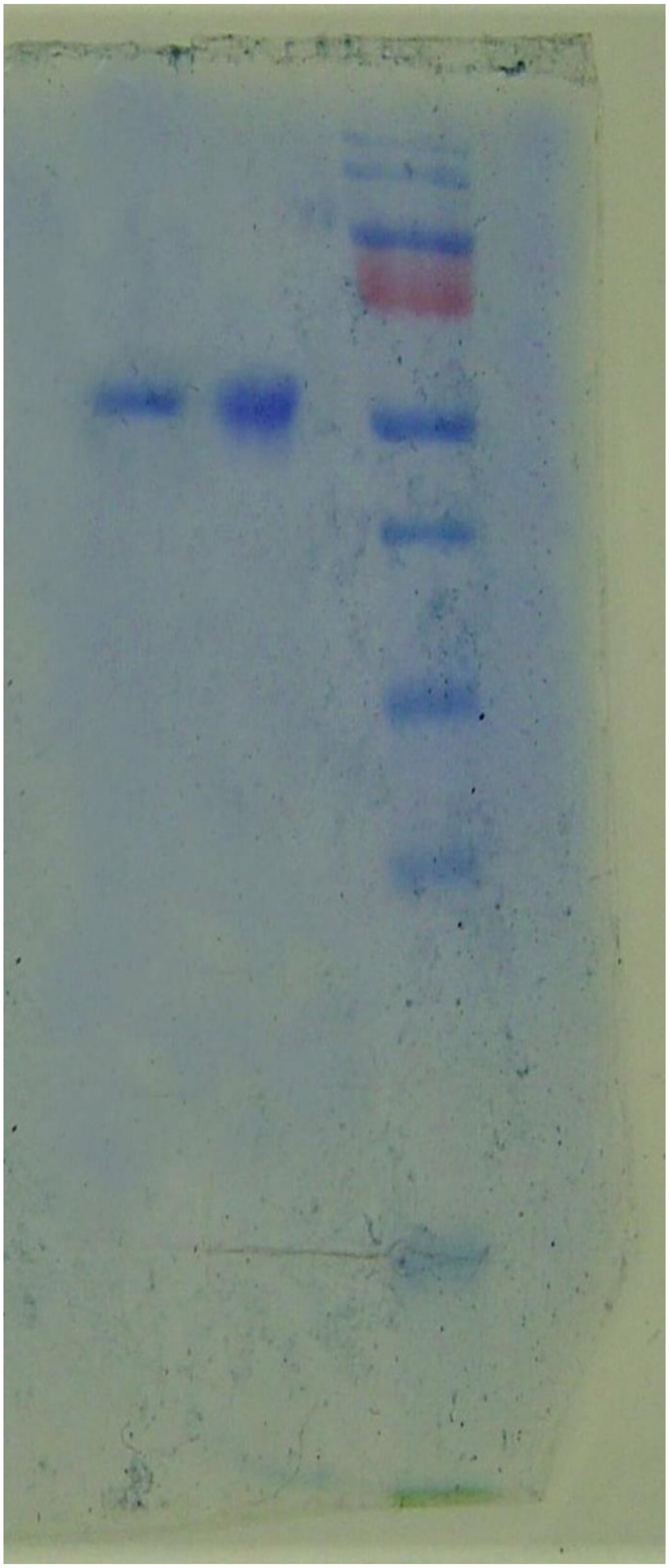

Supporting file (A). Determination of subunit molecular mass of glucoamylase (GA) produced by *A. oryzae* parent and mutant M-60(5).

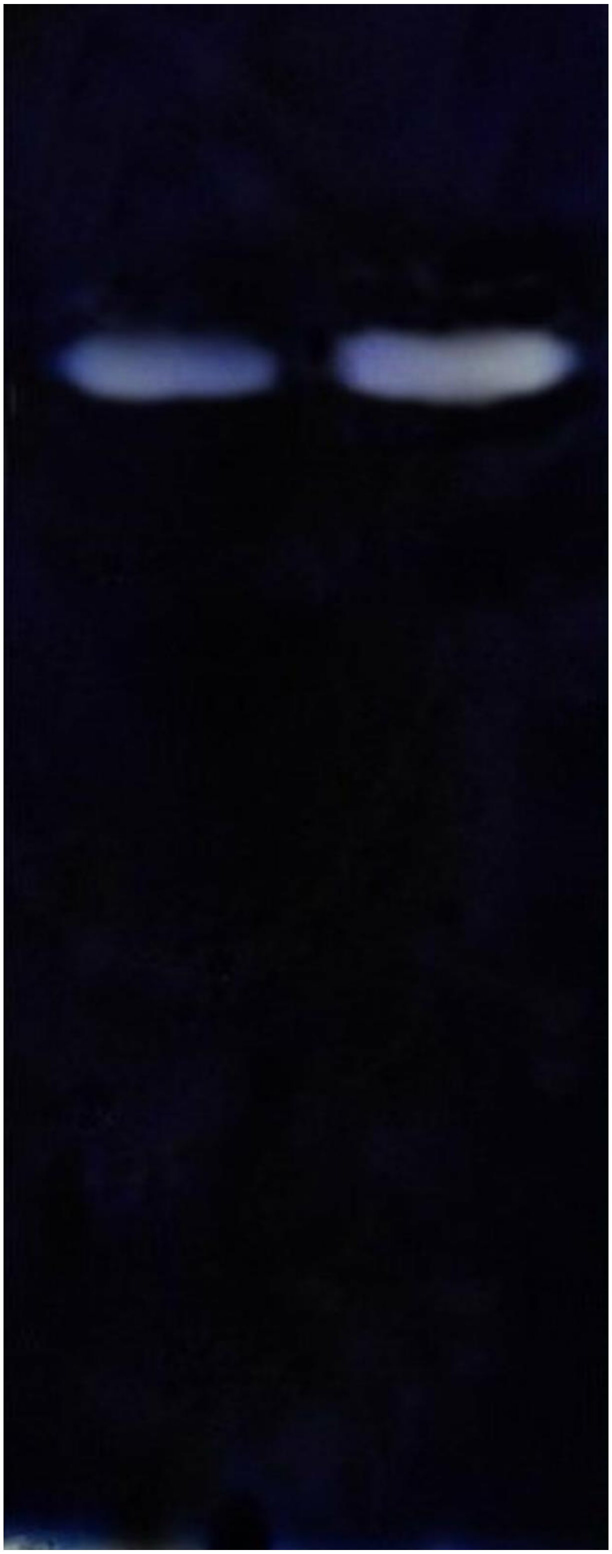

Supporting file (B). Determination of subunit molecular mass of glucoamylase (GA) produced by *A. oryzae* parent and mutant M-60(5).
