# Supplementary material for: Gama rays mediated improvement of catalytic efficiency and thermostability of glucoamylase by replacing active site leucine to isoleucene from super koji (Aspergillus oryzae)
Source: PLoS One. 2025 Apr 18;20(4):e0319261. doi: 10.1371/journal.pone.0319261 (PMC12007714; doi:10.1371/journal.pone.0319261)
Supplement: Attachment — Submitted filename: Response to Reviewers.pdf [file pone.0319261.s004.pdf]

Response to Reviewers

S. No	Reviewer (s)	Corr. Author Reply
**Reviewer # 1**		
INTRODUCTION<br>The introduction part clearly summarizes the current state of the topic and addresses the limitations of current knowledge in this field with clear and appropriate research question. However, the following minor changes need to addressed by authors;		
1	Line 44: Comma missing after 'However'.	Comma is added after 'However'. L 44
2	Line 44-45: Replace the sentence 'However their major short coming of low catalytic activity lead to consume high energy and restrictions in starch processing and fermentation' with 'However, their lower catalytic activity leads to consume high energy and restrictions in starch processing and fermentation'.	The sentence is being replaced. L 44-45
3	Line 61-63: Add reference (s) for the sentence 'Random mutagenesis induced by γ-rays may contribute in enhancement of industrial enzymes production; hence γ-ray mediated mutagenesis of A. niger made the mutant GA highly efficient in substrate hydrolysis'.	Reference is added. L 66
	Line 65: Comma missing after 'Previously'.	Comma is added after 'Previously'. L 68
	Line 71: Remove the 'data not shown'	The 'data not shown' is being removed. L 74
MATERIALS AND METHODS<br>The study design and methods are appropriate for the research question with enough detail for each experiment. However, the following minor changes need to addressed by authors;		
	Line 110: Add the paragraph under 'In silico sequence analysis' to heading 'Sequencing and in-silico point mutation identification in GA gene'.	Paragraph is being added under heading 'Sequencing and in-silico point mutation identification in GA gene'. L 133-140
	Line 113-112: Add full name for first time to 'EMBOSS, EMBL-EBI and NCBI'.	Full names for 'EMBOSS, EMBL-EBI and NCBI'are added. 134-137
	Line 139: Remove 's' from GA.	The 's' is being removed. L 153
RESULTS		

<table>
<tr><td colspan="3">The authors presented results clearly and accurately and all the relevant data been included as well as the data described in the text consistent with the data in the figures and tables. However, the following minor changes need to addressed by authors;</td></tr>
<tr><td></td><td>Line 205-206: Replace the sentence ‘DNA Extraction and PCR amplification of A. oryzae Mutant M-60(5) and parent strain indicated the size of genomic DNA and cDNA of GA genes as 2,039 bp and 2,241 bp, respectively’ with ‘The sequenced data analysis revealed the size of genomic DNA and cDNA of GA genes as 2,039 bp and 2,241 bp, respectively’.</td><td>The sentence is being replaced. L 219-222</td></tr>
<tr><td></td><td>Line 213: Incorrect abbreviations for A. Awamori (AA), A. nigar (AN), A. oryzae (AO). Write the correct abbreviations.</td><td>The abbreviations are being corrected. L 219-230</td></tr>
<tr><td></td><td>Line 255: Replace the word ‘consider’ with ‘considered’.</td><td>The word ‘consider’ is being replaced with ‘considered’. L 271.</td></tr>
<tr><td></td><td>Line 267: Replace the word ‘consider’ with ‘considered’.</td><td>The word ‘consider’ is being replaced with ‘considered’. L 283.</td></tr>
<tr><td colspan="3">DISCUSSION
The authors logically explained the findings and compared the findings with current findings in the research field. However, the following minor changes need to addressed by authors;</td></tr>
<tr><td></td><td>Line 351: Replace the word ‘may’ with ‘might’.</td><td>The word ‘may’ is being replaced with ‘might’. L 367.</td></tr>
<tr><td></td><td>Line 352-354: Replace the sentence ‘In the current study, it is supposed that the improvement in catalytic efficiency of GAs in mutant was linked with the change in a Leu203 to Ile203 at active site of GA Mutant M-60(5)’ with ‘we believe that improvement in catalytic efficiency of GAs in mutant was linked with the change in a Leu203 to Ile203 at active site of GA Mutant M-60(5)’.</td><td>The sentence is being replaced. L 368-371.</td></tr>
<tr><td></td><td>Line 391-392: The sentence ‘that shows decline in optimal activity above 50 °C, pH 5.5’ should be written as ‘which showed a decline in optimal activity above 50 °C, pH 5.5’.</td><td>The sentence is being replaced. L 408-410.</td></tr>
<tr><td></td><td>Line 419-421: Replace the sentence ‘Similarly, previously we had reported about the improvement in kinetic properties due to γ-rays mutagenesis on thermodynamics of starch hydrolysis’ with ‘Similarly, we already reported γ-rays mutagenesis-based improvement in kinetic properties on thermodynamics of starch hydrolysis’.</td><td>The sentence is being replaced. L 440-437.</td></tr>
<tr><td colspan="3">CONCLUSION
The authors logically concluded the whole research work but needs to be addressed the following minor changes.</td></tr>
</table>

	Line 441: Remove the word ‘drastic’.	The ‘drastic’ is being removed. L 461
	Line 446: Replace the word ‘may’ with ‘might’.	The word ‘may’ is being replaced with ‘might’. L 47.
**Reviewer # 2**		
1	L 43. Use full name for the first time e.g. ‘R. delemar’.	Full name is added. L 43
2	L 47. Replace word ‘their’ with ‘its’.	The word ‘their’ is being replaced with ‘its’. L 47.
3	L 49. The sentence ‘In recent era, strain improvement by site directed and random mutagenesis of fungi is an interesting research topic’ should be replaced with ‘In recent era, strain improvement by site directed and random mutagenesis of fungi is being used widely for industrial applications’.	The sentence is being replaced. L 50-53.
4	L 59. Use short name for ‘Aspergillus oryzae’. Same consistency must be kept throughout the manuscript.	Correction is being made. L 62
5	L 65-67. Support your claim by providing reference (if published) for ‘Previously we developed novel Aspergillus oryzae mutants by γ-rays’ mutagenesis for the enhanced production of thermostable α-amylases, which were also highly specific in the α-amylase production’.	Reference is being provided. L 70
6	L 72. Change the sentence ‘Novelty of current report is as it for the first time explains about effect of point mutation’ with ‘The current study reports for the first time effect of point mutation’.	The sentence is being changed. L 75-76
7	L 78. The word ‘Maintenance’ should be started with small letter.	Correction is being made. L 82
8	L 95. Ether use ‘hours’ or ‘hrs’. Correct the same in entire manuscript.	Corrections are being made. L 99, 100
9	L 98. Mention the pore size of muslin cloth.	The pore size is mentioned. L 102
10	L 99. Which instrument was used for lyophilization? Provide model and manufacturer name etc.	The model and manufacturer name are provided. L 103-104
	L 211. Add comma after ‘nucleotide position 703’.	Comma is added. L 226
	L 289. Correct the ‘pointed out’ to ‘pointed’.	Correction is being made. L 305
	L 289. Correct the ‘does not’ to ‘did not’.	Correction is being made. L 336
	L 344. Add comma after ‘nucleotide position 703’.	Comma is added. L 360
	L 360. Correct the ‘Similar’ to ‘similar’.	Correction is being made. L 376
	L 360. The word ‘Parent’ should be started with small letter.	Correction is being made. L 377

	L 380. Add comma after 'Similar findings regarding proton donating residues were presented by [15]'.	Comma is added. L 397
	L 386-388. This sentence is ambiguous and needs to be re-phrased 'Dehareng and Dive [47] evaluated the ionization energies (IE) as a function of the conformation for α-L-amino acids and three to five conformations for the arginine, lysine, isoleucine, tyrosine and tryptophan were optimized'.	The sentence is being rephrased and written clear for making sense. L 403-405
	L 390. Add comma after 'with optimum temp range of 45–55 °C (~80% of optimal activity)'.	Comma is added. L 407
	L 396-400. This sentence is ambiguous, long and needs to be re-phrased 'The Km of mutant M-60(5) GA for soluble starch hydrolysis at 50 °C, pH 5.0 was lower (0.06 mg ml-1) than the parental GA having 0.17 mg ml-1. Which is highly efficient than the already reported km values of naïve and modified enzyme i.e. 0.34 and 0.29 respectively and 2.1mg/ml of glucoamylase GA-LZ2'.	The sentence is being rephrased and written clear for making sense. L 414-418
	L 431. Add comma after 'strain was 274 kJ mol-1'.	Comma is added. L 451
	L 432. Add comma after 'mutant was as 237.02 kJ mol-1'.	Comma is added. L 452
	L 434. Add comma after 'mutant was 407.90 J mol-1K-1'.	Comma is added. L 454
