# Supplementary material for: Gama rays mediated improvement of catalytic efficiency and thermostability of glucoamylase by replacing active site leucine to isoleucene from super koji (Aspergillus oryzae)
Source: PLoS One. 2025 Apr 18;20(4):e0319261. doi: 10.1371/journal.pone.0319261 (PMC12007714; doi:10.1371/journal.pone.0319261)
Supplement: Attachment — Submitted filename: Comments to Authors_PONE-D-24-53783.docx [file pone.0319261.s002.docx]

The authors mutated *Aspergillus oryzae*, also known as Super Koji, by gama rays for enhancing catalytic efficiency and thermostability of glucoamylase by replacing active site leucine to isoleucene. The point mutation altered the conformation of the glucoamylase active site that contributed to improve the functional energy, resulted the stabilization of transition state which made it thermostable and highly efficient in starch hydrolysis. I would like to recommend the research work to be published in PLOS ONE. However, the following minor suggestions should be addressed by authors before publication.

L 43. Use full name for the first time e.g. ‘*R. delemar*’.

L 47. Replace word ‘their’ with ‘its’.

L 49. The sentence ‘In recent era, strain improvement by site directed and random mutagenesis of fungi is an interesting research topic’ should be replaced with ‘In recent era, strain improvement by site directed and random mutagenesis of fungi is being used widely for industrial applications’.

L 59. Use short name for ‘*Aspergillus oryzae*’. Same consistency must be kept throughout the manuscript.

L 65-67. Support your claim by providing reference (if published) for ‘Previously we developed novel *Aspergillus oryzae* mutants by γ-rays’ mutagenesis for the enhanced production of thermostable α-amylases, which were also highly specific in the α-amylase production’.

L 72. Change the sentence ‘Novelty of current report is as it for the first time explains about effect of point mutation’ with ‘The current study reports for the first time effect of point mutation’.

L 78. The word ‘Maintenance’ should be started with small letter.

L 95. Ether use ‘hours’ or ‘hrs’. Correct the same in entire manuscript.

L 98. Mention the pore size of muslin cloth.

L 99. Which instrument was used for lyophilization? Provide model and manufacturer name etc.

L 135. Add brief procedure detail to Protein assay.

L 211. Add comma after ‘nucleotide position 703’.

L 289. Correct the ‘pointed out’ to ‘pointed’.

L 289. Correct the ‘does not’ to ‘did not’.

L 344. Add comma after ‘nucleotide position 703’.

L 360. Correct the ‘Similar’ to ‘similar’.

L 360. The word ‘Parent’ should be started with small letter.

L 380. Add comma after ‘Similar findings regarding proton donating residues were presented by [15]’.

L 386-388. This sentence is ambiguous and needs to be re-phrased ‘Dehareng and Dive [47] evaluated the ionization energies (IE) as a function of the conformation for α-L-amino acids and three to five conformations for the arginine, lysine, isoleucine, tyrosine and tryptophan were optimized’.

L 390. Add comma after ‘with optimum temp range of 45–55 °C (~80% of optimal activity)’.

L 396-400. This sentence is ambiguous, long and needs to be re-phrased ‘The Km of mutant M-60(5) GA for soluble starch hydrolysis at 50 °C, pH 5.0 was lower (0.06 mg ml-1) than the parental GA having 0.17 mg ml-1. Which is highly efficient than the already reported km values of naïve and modified enzyme i.e 0.34 and 0.29 respectively and 2.1mg/ml of glucoamylase GA-LZ2’.

L 431. Add comma after ‘strain was 274 kJ mol^-1^’.

L 432. Add comma after ‘mutant was as 237.02 kJ mol^-1^’.

L 434. Add comma after ‘mutant was 407.90 J mol-1K^-1^’.
